# Therapeutic Potential of Nano-Sustained-Release Factors for Bone Scaffolds

**DOI:** 10.3390/jfb16040136

**Published:** 2025-04-09

**Authors:** Haoran Jiang, Meng Zhang, Yang Qu, Bohan Xing, Bojiang Wang, Yanqun Liu, Peixun Zhang

**Affiliations:** 1 Department of Orthopedics and Trauma, Peking University People’s Hospital, Beijing 100044, China; 2211110413@stu.pku.edu.cn (H.J.); mengzh2008@bjmu.edu.cn (M.Z.); 2311110437@stu.pku.edu.cn (Y.Q.); 2411210321@bjmu.edu.cn (B.X.); wangbojiang@qdu.edu.cn (B.W.); 2 Department of Trauma & Orthopedics, Peking University People’s Hospital Qingdao Hospital, Qingdao 266111, China; 3 National Centre for Trauma Medicine, Beijing 100044, China; 4 Key Laboratory of Trauma and Neural Regeneration, Peking University, Beijing 100044, China; 5 Beijing Laboratory of Trauma and Nerve Regeneration, Peking University, Beijing 100044, China; 6 Department of Orthopedic Surgery, Yanbian University Hospital, 1327 Juzi St., Yanji 133002, China

**Keywords:** sustained release, nanotechnology, bone graft, tissue engineering, clinical trial

## Abstract

Research on nano-sustained-release factors for bone tissue scaffolds has significantly promoted the precision and efficiency of bone-defect repair by integrating biomaterials science, nanotechnology, and regenerative medicine. Current research focuses on developing multifunctional scaffold materials and intelligent controlled-release systems to optimize the spatiotemporal release characteristics of growth factors, drugs, and genes. Nano slow-release bone scaffolds integrate nano slow-release factors, which are loaded with growth factors, drugs, genes, etc., with bone scaffolds, which can significantly improve the efficiency of bone repair. In addition, these drug-loading systems have also been extended to the fields of anti-infection and anti-tumor. However, the problem of heterotopic ossification caused by high doses has led to a shift in research towards a low-dose multi-factor synergistic strategy. Multiple Phase II clinical trials are currently ongoing, evaluating the efficacy and safety of nano-hydroxyapatite scaffolds. Despite significant progress, this field still faces a series of challenges: the immunity risks of the long-term retention of nanomaterials, the precise matching of multi-factor release kinetics, and the limitations of the large-scale production of personalized scaffolds. Future development directions in this area include the development of responsive sustained-release systems, biomimetic sequential release design, the more precise regeneration of injury sites through a combination of gene-editing technology and self-assembled nanomaterials, and precise drug loading and sustained release through microfluidic and bioprinting technologies to reduce the manufacturing cost of bone scaffolds. The progress of these bone scaffolds has gradually changed bone repair from morphology-matched filling regeneration to functional recovery, making the clinical transformation of bone scaffolds safer and more universal.

## 1. Introduction

Bone-tissue engineering represents a crucial field within regenerative medicine [1]. Specifically, its core target is to repair or reconstruct various types of bone defects. This is achieved through the synergy of biomaterials, cells, and growth factors. It is the integration of nano-sustained-release factors, which load growth factors [2], drugs [3], genes [4], etc., with bone scaffolds that have led to a remarkable enhancement in the efficiency of bone repair.

Diseases like osteoporosis and infection can trigger the non-union of bones after fractures, thereby giving rise to bone defects [5]. To cope with these issues related to bone defects, a series of bone scaffolds have been developed for the purpose of repairing these defects [6]. In recent years, bone scaffolds created using a combination of biological scaffolds and nano-sustained-release factors have experienced substantial progress. However, there are wide variations in manufacturing standards across different processes and the properties of these scaffolds also show significant disparities. We have systematically scrutinized relevant papers on the application of nano-sustained-release factors in bone scaffolds over recent decades and have carried out an in-depth literature review. This review comprehensively summarizes the fundamental properties of bone scaffolds integrated with nano-sustained-release factors, several fabrication methods for bone scaffolds, and their properties and characteristics, with a particular focus on the properties associated with factor release. Moreover, this review also systematically sums up the current research status, the challenges encountered, and promising future research directions in the application of relevant bone scaffolds. On the premise of covering materials science and basic medicine, this paper focuses more on the expectations for the clinical application of materials, including the characteristics required by surgeons and potential risks and challenges.

## 2. Materials and Methods

### 2.1. Attribute Requirements for Bone Scaffolds

Bone is an important component of the human skeletal system. It provides structural support for the human body, enables movement by facilitating muscle contraction, and also serves as a vital reservoir for minerals in the body [7]. The normal structure of bone is similar to that of other connective tissues, being composed of the following three components: cells, fibers, and a matrix [8,9,10]. However, the extracellular matrix of bone tissue contains a large amount of calcium salt deposits, which maintain the relative stability of calcium content in body fluids. The calcium salts in bone tissue are relatively insoluble, especially phosphates and carbonates. Phosphate can dissolve in the extracellular fluid at a concentration of 10^−3^ mol/L. Meanwhile, the hydroxyapatite crystals in bone tissue dissolve in the extracellular fluid in the form of Ca^2+^ and HPO_4_^2−^, and these ions attach to the bone crystals.

Bone is an important part of the body’s weight-bearing structure and also comprises a dynamic system. Under physiological conditions, it has the ability to remodel, remove, and replace itself simultaneously [11]. Thanks to this self-remodeling ability, the ultimate result of fracture healing is the regeneration of normal bone structure. Moreover, when there is a small bone defect, bone tissue can heal itself. However, if there is a large bone defect, or if the patient has some underlying diseases such as diabetes, obesity, or is a smoker, the normal healing process may be hindered, resulting in the non-union or delayed union of bones [12]. The conventional clinical treatment method for large-segment bone defects is bone grafting [13]. The vast majority of these are autologous bone grafts, and allogeneic bone or synthetic bone substitutes are also used [14]. Nevertheless, autologous bone grafting remains the gold standard for treating the non-union of bones [14]. Autologous cancellous bone is generally considered an ideal bone graft, mainly because it can provide osteoinductive growth factors such as BMP, osteoblasts, and osteogenic induction scaffolds, with a success rate of 50–80%. However, autologous bone grafting still has its limitations, including donor-site complications, limited availability, and the risk of infection.

In recent decades, the development of synthetic grafts as alternatives to bone grafts has been promoted, and some of them have been used in the clinical treatment of fractures [15]. These synthetic grafts are more easily obtainable compared to autologous cancellous bone and can be processed into different shapes to meet the requirements of specific orthopedic surgeries [16]. Additionally, during the continuous improvement of materials, these grafts have avoided the risk of disease transmission, and also possess beneficial mechanical properties, good biocompatibility, and a low risk of infection.

From the perspective of materials science, bone scaffolds need to possess a three-dimensional porous structure, biocompatibility, mechanical adaptability (with more stringent requirements for applications in weight-bearing bone regions), and controllable degradation [17,18]. The introduction of nanotechnology, on the one hand, optimizes the physicochemical properties of the scaffolds, ensuring that the physicochemical performance of bone scaffolds more closely meets these clinical needs [19]. On the other hand, nanotechnology can better achieve the precise carrying and controlled release of factors [20].

### 2.2. Scaffold Material Types

**Figure 1:**
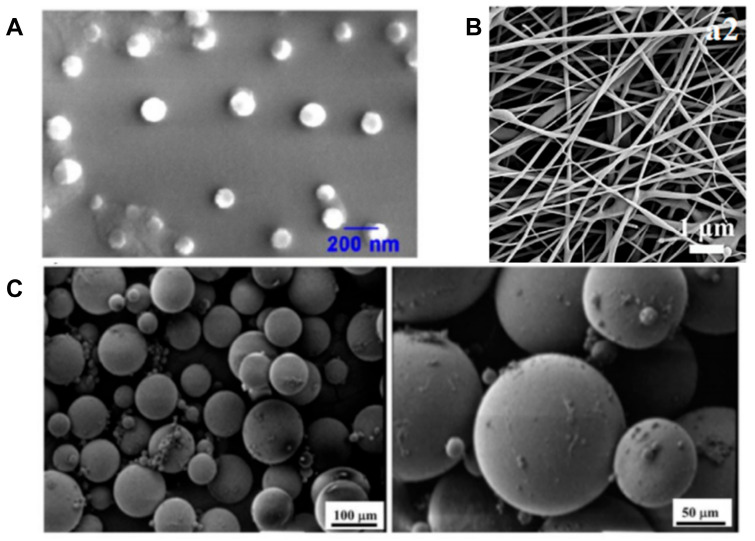
(**A**) SEM images of AHK-CaP/siRNA nanoparticles (NPs). Scale bars = 200 nm (Reprinted with permission from Ref. [20]. Copyright 2024 American Chemical Society); (**B**) SEM pictures of the surface of PCL/PCS (Reprinted with permission from Ref. [23]. Copyright 2017 Elsevier); (**C**) The scanning electron microscopy images show the surface of poly (lactic-co-glycolic acid) (PLGA)–alendronate (ALN) microspheres and indicate that the average size of the microspheres was approximately 100 μm (Reprinted with permission from Ref. [25]. Copyright 2022 Elsevier).

Scaffold material types are divided into organic materials and inorganic materials. Organic materials are further divided into natural polymer materials and synthetic polymer materials. Natural polymer materials include collagen, gelatin, chitosan, hyaluronic acid (Figure 1A), etc. [20,21]. These materials generally have good biocompatibility, but relatively weak mechanical properties. Therefore, they are more commonly used in the fabrication of non-weight-bearing bones or functional bone scaffolds and are generally less used in the fabrication of weight-bearing bone scaffolds. For example, a scaffold of grafted hydroxyapatite (g-HA) is used to simulate the natural bone matrix, and nanoparticles (such as PLGA) are combined to load BMP-2 to promote osteogenic differentiation [22]. Synthetic polymer materials include polylactic acid (PLA), polycaprolactone (PCL) (Figure 1B), polyethylene glycol (PEG), etc. [23,24]. Their artificial synthesis generally endows them with stronger mechanical strength than natural polymer materials. At the same time, artificial synthesis gives the materials better degradation properties and more controllability. For example, a PCL nanofiber scaffold (Figure 1C) is fabricated through electrospinning technology, and then VEGF is loaded through surface functionalization to achieve the synergy of angiogenesis and bone regeneration [25]. Among cytokines that promote bone regeneration, both BMP-2 and VEGF have been widely utilized [26,27]. BMP-2 directly induces osteogenic differentiation, while VEGF stimulates neovascularization (formation of new blood vessels). Although VEGF alone exhibits weaker osteogenic effects compared to BMP-2, their combined application demonstrates synergistic benefits. This strategy alleviates complications arising from inadequate blood supply in bone tissues when BMP-2 is used alone [28]. For instance, bone scaffolds co-delivering both factors have shown promising results in diabetic rat models with bone defects, enhancing both vascularization and bone repair efficiency [29]. It is crucial to note that PLGA (poly(lactic-co-glycolic acid)) and PCL (polycaprolactone) exhibit distinct material properties. On one hand, PCL’s longer degradation cycle enables the sustained release of loaded factors over extended periods, aligning with the prolonged requirements of bone regeneration [30]. On the other hand, PLGA degradation generates acidic byproducts (e.g., lactic and glycolic acids), which may trigger localized inflammatory responses or impair cell activity [31]. Therefore, in bone defect repair strategies involving co-delivery of dual factors (e.g., VEGF and BMP-2), PCL-based scaffolds hold greater promise due to their biocompatibility and degradation kinetics tailored for synergistic therapeutic outcomes [32].

Inorganic materials include hydroxyapatite (HA), β-tricalcium phosphate (β-TCP), and bioactive glass (BGs) [17,33,34]. Inorganic materials often have good physicochemical and mechanical properties, as well as osteoconductivity [35]. Some inorganic materials of calcium–magnesium phosphates can also provide raw materials for bone regeneration. For example, by integrating deferoxamine (DFO) into a mineralized collagen-based sodium alginate composite hydrogel and combining it with a new 3D-printed titanium-alloy microporous scaffold, the bioactivity of the implant can be enhanced and osseointegration can be promoted [36].

In these materials, a unique inorganic compound—hydroxyapatite (Ca_10_(PO_4_)_6_(OH)_2_) exhibits ion substitution phenomena [37]. This feature allows for structural and physicochemical modifications, significantly influencing the scaffold’s drug-loading capacity and release behavior. Hydroxyapatite possesses a crystal structure characterized by three replaceable sites: (1) calcium (Ca^2+^) sites that can be substituted by Sr^2+^, Mg^2+^, Zn^2+^, Fe^2+^/Fe^3+^, and Na^+^; (2) phosphate (PO_4_^3−^) sites that can be replaced by CO_3_^2−^ and SiO_4_^4−^; and (3) hydroxide (OH^−^) sites that can be substituted by F^−^, Cl^−^, and CO_3_^2−^ [38,39,40,41]. For instance, incorporating magnesium into the hydroxyapatite crystal lattice can induce distortion and displacement, thereby reducing crystallinity and enhancing solubility [42]. Furthermore, at different stages of bone differentiation, a certain amount of magnesium doping has been shown to exert beneficial effects on the differentiation of mesenchymal stem cells toward osteoblasts [43]. There is another interesting example: by performing a solubilization-sedimentation reaction on hydroxyapatite nanorods, researchers synthesized carbonate-substituted hydroxyapatite nanorods [44]. The scaffold exhibits high antibacterial activity, which can be further enhanced by adding fibrous nanofibers [45]. It is noteworthy that the calcium–phosphorus molar ratio of the synthesized material (1.66) is close to that of biological apatite (1.71) [46].

### 2.3. Preparation Techniques

**Figure 2:**
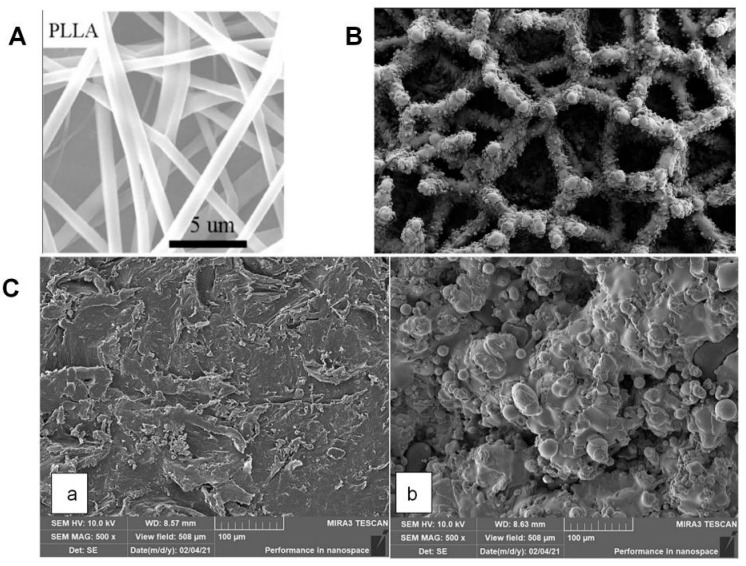
(**A**) ESEM images of electrospun nanofiber mats for poly(L-lactide) (PLLA) (Reprinted with permission from Ref. [47]. Copyright 2014 Elsevier); (**B**) Microscopic (at 61× magnification) depiction of the microstructure of the 3D-pTi interbody cage (Reprinted with permission from Ref. [48]. Copyright 2022 American Chemical Society); (**C**) SEM images of n-HA/PA66 (Reprinted with permission from [59]. Copyright 2021 Springer Nature) (**a**) and n-HA/PA66-PST (**b**). The two sample groups were examined using SEM. The surface of the n-HA/PA66 implants was relatively smooth and lacked surface features. The PST coating on the n-HA/PA66 sample demonstrates a rough topography for bone growth and cavities for bone ingrowth.

Following their development in recent years, the preparation techniques for nano-sustained-release bone scaffolds have become increasingly diverse, including methods such as electrospinning technology (Figure 2A), three-dimensional (3D) printing technology (Figure 2B), self-assembly technology, and nano-coating technology (Table 1) [47,48]. Fabricated bone scaffolds are also progressing towards clinical use.

Among these fabrication techniques, the most common and widely used one is electrospinning technology. Electrospinning can prepare fibers with diameters ranging from nanoscale to micron-scale by adjusting parameters such as voltage and solution concentration, forming high specific surface area and porous structure. Simultaneously, electrospinning can be applied to a variety of materials, including natural polymers and synthetic polymers, and can composite inorganic materials to optimize the mechanical strength and bioactivity of scaffolds. Through electrospinning, nanofibers (with a diameter of 50–500 nm) can be stably synthesized, and the high specific surface area of these nanofibers is conducive to factor loading [49,50,51]. On the one hand, nanofibers with smaller diameters are capable of loading a greater quantity of factors, due to their relatively large specific surface areas [52]. Moreover, through techniques like coating, the release profiles of these factors can be effectively modulated. On the other hand, an excessively small diameter will render the fibers more brittle. This is a critical aspect that demands careful equilibrium when manufacturing bone scaffolds, which have stringent mechanical property requirements. Simultaneously, in clinical settings, blood or exudates typically contain abundant proteins. In such cases, pores that are too minute will be occluded by these proteins, thereby impeding drug release. Consequently, when determining the diameter of nanofibers, it is essential to comprehensively consider multiple factors.

Three-dimensional (3D) printing technology refers to the construction of complex structures through fused deposition modeling (FDM) or digital light processing (DLP) technology, which are advantageous in terms of material adaptability, structural accuracy, functional design, etc., combined with microfluidic technology to achieve the gradient distribution of factors [53]. FDM technology is more suitable for printing bone scaffolds with high load requirements, especially for load-bearing bone [54]. By adjusting the printing path, layer height, and filling density (porosity), the mechanical properties of cancellous bone or cortical bone can be simulated. At the same time, this technique has low equipment costs, fewer material restrictions, and a faster printing speed, rendering it suitable for the preparation of large-size scaffolds. However, FDM technology also has limited resolution, produces high surface roughness, and reaches high temperatures. Alternatively, DLP can print micron-sized pores, cantilever structures, and even biomimetic vascular networks. It can also use hydrogels, bioceramics, etc., and can directly mix cells or growth factors into scaffolds. However, DLP technology still has the limitations of low mechanical strength, limited material selection, and high costs. Three-dimensional (3D) printing can customize the shape of the scaffold according to the image data of the bone defect to achieve accurate matching. Three-dimensional printing supports layer-by-layer printing of a variety of materials and can match the mechanical requirements of bone tissue by regulating the porosity and fiber arrangement direction of scaffolds [55]. It can also integrate vascular channels to promote nutrient delivery and neoangiogenesis. Self-assembly technology refers to the formation of ordered nanostructures using intermolecular forces [56]. Self-assembled materials can simulate the dynamic characteristics of natural ECM, enhance the biological functions of scaffolds, and can also integrate magnetic nanoparticles (such as SPIONs) or conductive materials into scaffolds, which can endow them with a magnetic response or electrical stimulation functions. For example, a polypeptide self-assembly scaffold can be used to load siRNA to inhibit bone resorption [57]. Nano-coating technology refers to the construction of a nano-scale sustained-release layer on the surface of the scaffold through coating techniques, for example, a plasma-sprayed HA coating and a layer-by-layer self-assembly (LbL) coating [58]. The former (Figure 2C) is suitable for metal implants, combining mechanical strength and bioactivity, but the process of loading heat-sensitive factors needs to be optimized [59]. The latter is suitable for natural or synthetic polymer scaffolds and achieves the sequential release of multiple factors, but the problem of coating stability needs to be solved [60]. The surface properties of LbL coating are quite different from those of traditional bone scaffold materials (such as titanium alloy, polymer, ceramics, etc.), which are easy to peel off and can easily inactivate factors during the manufacturing process. At the same time, LbL coating degrades rapidly, and using it to create a uniform cover on the surface of porous structures is difficult, which also increases the difficulty of applying LbL coating to national-scale model production. At present, the most promising solutions to this issue include multi-functional coatings, intelligent layers, and real-time monitoring coatings.

### 2.4. Sustained-Release Factors

By loading sustained-release factors on bone scaffolds, the scaffolds can be endowed with desired functions, including promoting bone formation and anti-infection. With the continuous development of nanotechnology, the types and functions of nano-sustained-release factors have also made significant progress. Generally, nano-sustained-release factors are divided into growth factors, small-molecule drugs, and gene-delivery systems. In the application of tissue-engineering scaffolds, growth factors are widely used in various bone scaffolds due to their ability to directionally promote or inhibit tissue growth.

#### 2.4.1. Bone Morphogenetic Proteins

**Figure 3:**
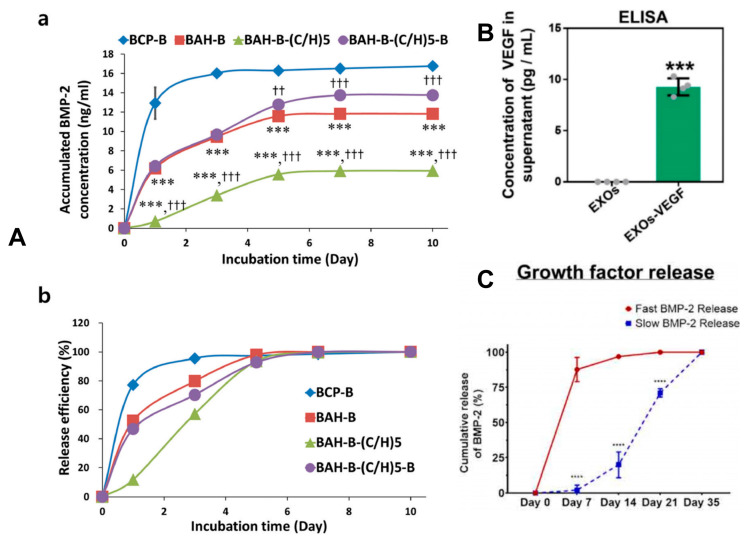
(**A**) BMP-2 release profile according to each sample (Reprinted with permission from Ref. [61]. Copyright 2021 Elsevier); BMP-2 in vitro (**a**) release behaviors and (**b**) release efficiency from BMP-2-loaded BCP (BCP–B), heparin-conjugated A-PSQ/BCP with BMP-2 (BAH-B), (C/H)5 layer by layer film-coated BAH-B (BAH-B-(C/H)5), and BMP-2-loaded BAH-B-(C/H)5 (BAH-B-(C/H)5–B) in PBS (pH = 7.4). *** indicate “*p* < 0.001”, compared to BCP-B. ††, and ††† indicate “*p* < 0.01” and “*p* < 0.001”, respectively compared to BAH-H. (**B**) Both qRT-PCR and ELISA clearly confirmed that the relative expression of VEGF in EXOs-VEGF was significantly higher than that in pure EXOs (Reprinted from Ref. [66]); (**C**) Cumulative release of BMP-2 of the fast-release bioink versus the slow-release bioink (Reprinted from Ref. [68]). **** indicate “*p* ≤ 0.0001”.

Bone morphogenetic proteins (BMPs), specifically BMP-2 (Figure 3A) and BMP-7, have been approved for clinical use in spinal fusion and fracture repair [61,62]. They are not only used in combination with bone scaffolds but also have independent application scenarios [63]. However, high doses can easily cause heterotopic ossification and inflammation [64]. Therefore, controlled release is required, and simple high-dose continuous sustained release is not feasible. This has led to corresponding nano-sustained-release strategies. For example, PLGA microspheres are used to load BMP-2, achieving a sustained release of up to 4 weeks in the scaffold, and the dose administered per unit time is reduced to 1/10 of the traditional method [65]. Another commonly used growth factor is vascular endothelial growth factor (VEGF) (Figure 3B), which can promote angiogenesis and is often used in combination with BMPs [66,67]. Methylcellulose/RGD γ-irradiated alginate nanoparticles (Figure 3C) co-load VEGF and BMP-2 to achieve spatiotemporal sequential release (VEGF is released early, and BMP-2 is released later) [68]. There are also common growth factors such as fibroblast growth factor (FGF), which can promote the proliferation of mesenchymal stem cells (MSCs) [69].

#### 2.4.2. Small-Molecule Drugs

Common small-molecule drugs used for nano-sustained-release in bone scaffolds include antibiotics, anti-inflammatory drugs, and anti-osteoporosis drugs. Antibiotics are widely used, such as gentamicin, mainly for contaminated wounds or post-operative anti-infection, often loaded with nano-silver or silica [70]. Anti-inflammatory drugs such as dexamethasone can regulate the immune micro-environment and inhibit fibrosis [71].

#### 2.4.3. Anti-Osteoporosis Drug

The representative anti-osteoporosis drug is bisphosphonates such as alendronate sodium, which mainly reduces bone resorption by inhibiting the activity of osteoclasts, thereby achieving an anti-osteoporosis effect [72].

However, traditional growth factors have obvious limitations. On the one hand, the half-life of growth factors is short, making it difficult to maintain an effective concentration with a single dose. On the other hand, multiple doses or continuous sustained release can risk of overdose. Additionally, growth factors are expensive and prone to side effects; for example, BMPs are likely to cause heterotopic ossification [73]. Gene delivery technology delivers the target gene into the nucleus of target cells through a gene vector and uses the cell’s own protein-synthesis mechanism to express functional proteins [74]. In this way, by delivering genes encoding target proteins (such as BMP-2 and VEGF) to target cells (such as mesenchymal stem cells and osteoblasts), the cells can continuously express functional proteins (for weeks to months), significantly reducing the dose requirement and achieving precise regulation. According to the vectors of the gene delivery system, it can be divided into two categories, including non-viral vectors, such as cationic polymers (PEI, etc.), liposomes, or nanodiamonds, and viral vectors, such as adenoviruses or lentiviruses [75,76]. Although viral vectors are highly efficient, the potential risk of viral infection following their use has led to significant controversy over their safety. There have been previous reports of PEI-modified mesoporous silica nanoparticles, delivering the Runx2 gene to promote the osteogenic differentiation of MSCs [77].

#### 2.4.4. Detection Method of Drug Release

The detection methods for drug release can be categorized into in vitro and in vivo assays. Bone scaffolds loaded with nanosized sustained-release factors require precise control over both release duration and drug concentration, necessitating a combination of multiple detection methods rather than relying on a single approach.

The most widely used in vitro method is the drug release experiment, where drug-loaded scaffolds or microspheres are immersed in simulated physiological media (e.g., phosphate-buffered saline, PBS) [78]. Drug concentrations are periodically sampled and analyzed to calculate the cumulative release rate. This method is simple, convenient, and cost-effective. Other in vitro methods include dynamic dialysis, which evaluates long-term release kinetics under simulated dynamic physiological conditions.

Compared to the low cost and simplicity of in vitro assays, the primary advantage of in vivo detection lies in its ability to directly capture the release kinetics, metabolic pathways, and biological effects of drugs under real physiological conditions. For example, drug-loaded scaffolds are implanted into bone defect sites in animal models (e.g., rats or rabbits), followed by periodic collection of blood or tissue samples to measure drug concentrations and metabolites [79]. This approach is straightforward and reliable, with extensive applications in preclinical studies. Other in vivo methods include microdialysis (continuous sampling of interstitial fluid near the implant site) and imaging-assisted live tracking techniques (e.g., fluorescence labeling or MRI-based monitoring).

Traditional detection methods are no longer sufficient to meet the demands of modern bone scaffold development. Current research predominantly employs integrated strategies that combine in vitro drug release experiments with animal model implantation to comprehensively evaluate the performance of sustained-release systems. The specific application of these methods should be flexibly tailored to research objectives (e.g., drug type, scaffold material, expected release duration), with a strong emphasis on complementary validation between in vitro and in vivo data to ensure robustness and clinical translatability.

### 2.5. Sustained-Release Mechanisms and Controlled-Release Strategies

In bone tissue engineering, the choice of nanocarriers directly affects the release kinetics and bioavailability of growth factors, drugs, or genes. Therefore, when fabricating nano-sustained-release bone scaffolds, it is necessary to comprehensively consider and carefully select the correct type of carrier, based on its material properties and application scenarios. The following systematically analyzes the main types of nanocarriers and their controlled-release mechanisms.

#### 2.5.1. Liposomes

**Figure 4:**
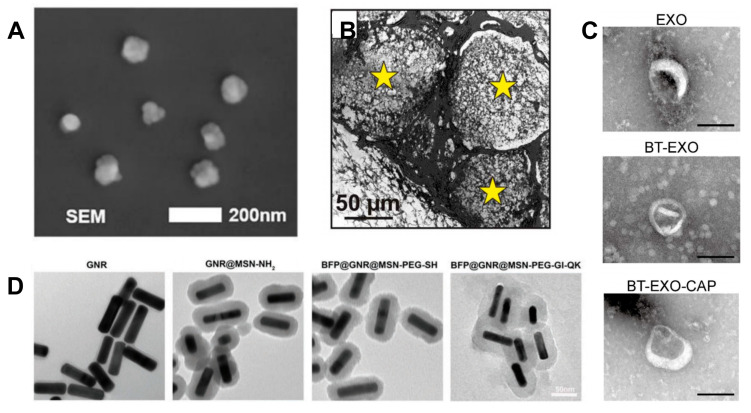
(**A**) The SEM image of lipid nanoparticles (LNPs)-siRNA/GM. Scale bar = 200 nm (Reprinted from Ref. [81]); (**B**) The cross-section of the collagen scaffolds is examined via SEM, and exhibits the presence of MCs (stars) within the collagen matrix (Reprinted with permission from Ref. [84]. Copyright 2015 John Wiley and Sons); (**C**) Representative TEM images of EXOs, BT-EXOs, and BT-EXO-CAP (Reprinted with permission from Ref. [93]. Copyright 2023 Springer Nature); (**D**) Transmission electron microscopy (TEM) images of different nanoparticles (GNR, GNR@MSN-NH2, BFP@GNR@MSN-PEG-SH, and BFP@GNR@MSN-PEG-GI-QK) (Reprinted with permission from Ref. [88]. Copyright 2024 John Wiley and Sons).

Liposomes are composed of a phospholipid bilayer, forming a hydrophilic core and a hydrophobic interlayer [80]. They achieve targeted delivery through a biomimetic membrane structure and can simultaneously load water-soluble molecules (such as BMP-2) and fat-soluble drugs (such as bisphosphonates). Liposomes have two release mechanisms. One is passive diffusion, where small-molecule drugs (such as gentamicin) are released through the diffusion of the phospholipid membrane. The other is the fusion of liposomes with the cell membrane, directly releasing the contents into the cell membrane. For example, in the case of siRNA delivery, liposomes (Figure 4A) are an environment-responsive carrier [81]. For instance, by modifying liposomes with pH-sensitive phospholipids (such as DOPE), rapid release can occur in an acidic environment (pH 6.5–6.8, such as in the inflammatory region) due to the instability of the membrane structure [82]. Previous research reported that liposomes loaded with alendronate sodium were conjugated with RGD peptides on the surface, targeting the integrin αvβ3 receptor of osteoclasts, which could triple the efficiency of integrin-receptor-mediated inhibition of bone resorption (producing a 70% reduction in osteoclast activity in vitro) [83]. However, the structure of liposomes itself is not stable. Phospholipids are prone to oxidation and degradation, resulting in poor stability. They cannot be simply stored for a long time and need to be stored via lyophilization, which increases the cost of storage and use.

#### 2.5.2. Polymer Nanoparticles (Such as PLGA, PCL, etc.)

Polymer nanoparticles can be artificially controlled to change their properties of degradation and sustained release, enabling the achievement of long-term release. For example, PLGA (poly(lactic-co-glycolic acid)) can adjust the ratio of lactic acid/glycolic acid (for example, to 50:50 or 75:25, with degradation cycles corresponding to 2 weeks and 6 months, respectively). Another example comprises a novel dual-growth-factor delivery system that is composed of poly(lactic-co-glycolic acid) (PLGA) nanoparticles (NP) and sodium alginate microcapsules (MC). The PLGA NPs and sodium alginate MCs (Figure 4B) were used to load bone morphogenetic protein (BMP)-2 and vascular endothelial growth factor (VEGF), respectively, using the electro-dripping method [84].

Another widely used artificial polymer is PCL, polycaprolactone. It has strong hydrophobicity and a degradation time of more than 12 months, making it suitable for the repair of weight-bearing bones that require long-term mechanical support [85]. At the same time, polymers can achieve biphasic drug release. In the early stage, drugs are released through surface pores, and in the later stage, they are released through polymer degradation. By using a porogen or coating the surface of the scaffold, the release rate can be further adjusted. For example, when PLGA layer-by-layer (LbL) films were used to load BMP-2 (0.5 μg) and implanted into rat cranial defects, the release rate was over 90% after 28 days, and the volume of new bone was twice that of the direct-injection group [60]. However, lactic acid is an acidic degradation product that may cause local inflammation. For the purpose of not affecting the pH of the microenvironment, some alkaline materials are often required to be compounded in the materials to neutralize lactic acid.

#### 2.5.3. Mesoporous Materials

Mesoporous materials are often used in controlled-release systems due to their high drug-loading capacity and precise controlled-release properties. For example, the pore size of mesoporous silica (MSNs) is 2–10 nm, the specific surface area is greater than 900 m^2^/g, and the drug-loading capacity can reach 30% by mass [86]. Another application method is to combine responsive degradation and controlled release. For example, ZIF-8 (a zinc–imidazole framework) has been used to fabricate metal–organic frameworks, which can achieve antibiotic-controlled releases for infected bone defects through pH-responsive degradation [87]. For this type of mesoporous material, the pore size of the bone scaffold generally needs to be selected according to the drug. Usually, the pore size is chosen to be more than 1.5 times the diameter of the drug molecule, to avoid blockage and ensure drug release. In addition to loading drugs through pores, mesoporous materials can also be surface-functionalized. For example, the surface of mesoporous silica is modified with amino groups (Figure 4D), and the loading efficiency of negatively charged drugs (such as DNA) is enhanced through electrostatic adsorption [88]. This not only increases the drug-loading efficiency but also enables sequential release. For example, previous research loaded the inner layer of MSNs with BMP-2 (sustained release for 28 days), coated the outer layer with sodium alginate gel, and encapsulated it with VEGF (rapid release for 7 days), which shortened the time taken for vascularized bone regeneration in rabbit femoral defects by 40% [89]. However, silica-based materials do not degrade, which greatly limits the application of mesoporous materials. The long-term retention of non-degradable materials in the body may cause tissue fibrosis and tissue inflammation. Further widespread application requires the development of biodegradable silica-based materials.

#### 2.5.4. Exosomes

In addition to these conventional carriers, the intelligent delivery of exosomes—natural vesicles—has received increasing attention in recent years. Exosomes have a diameter of 30–150 nm and are natural vesicles secreted by cells, carrying proteins, miRNAs, and lipids from the parent cells [90]. Their advantages include significantly low immunogenicity and targeted homing ability (for example, MSCs-derived exosomes tend to accumulate at bone injury sites). Researchers engineer exosomes to carry drugs [91]. For example, BMP-2 mRNA was loaded into exosomes using electroporation or sonication, and after transfection into cells, osteogenic proteins were continuously expressed (>14 days) [92]. There are also methods to modify the exosome membrane through genetic engineering techniques (Figure 4C), expressing targeted peptides (such as SDSSD) on the exosome membrane to specifically bind to bone matrix collagen [93]. According to relevant reports, the application of exosomes loaded with anti-miR-214 to inhibit osteoclast differentiation can increase the bone density of mice by 25% in a mouse model [94]. Due to their extremely small diameter, exosomes can cross the blood–bone barrier, making them suitable for systemic administration to treat systemic bone diseases.

#### 2.5.5. Strategies for Growth Factor Binding and Release Mechanisms

In general, the factor-binding strategies for nanosized sustained-release bone scaffolds must balance loading capacity, release efficiency, and preservation of factor bioactivity. Among these strategies, physical adsorption is the most commonly used method [95]. This involves attaching growth factors to the scaffold surface or within its pores through electrostatic interactions, hydrophobic forces, or hydrogen bonding. While this approach is simple to implement and preserves factor activity, it often fails to achieve long-term sustained release, as adsorbed factors are prone to rapid initial burst release. Chemical conjugation is another widely used drug-loading method. This approach covalently immobilizes factors onto functional groups of scaffold materials, offering highly controllable drug release [96]. However, its applicability is severely restricted by the limited compatibility between specific factors and scaffold chemistries. Additionally, the conjugation process may compromise factor bioactivity if reaction conditions are not rigorously optimized. A highly promising alternative is nanocarrier encapsulation, where factors are encapsulated within microspheres or nanoparticles, which are then embedded into the scaffold matrix [97]. This method provides enhanced stability and tunable release kinetics. In the context of bone scaffolds, biomimetic mineralization-based binding offers a unique strategy. By leveraging the ion adsorption capacity of nano-hydroxyapatite, factors are embedded within the mineralized coating of the scaffold [98]. This enables the synchronized release of factors with nano-hydroxyapatite degradation, closely matching the timeline of bone regeneration. However, the drug-loading capacity is inherently limited by the thickness and uniformity of the mineralized layer, requiring precise control over deposition parameters.

The release kinetics of factors are governed by the interplay of material properties, loading strategies, and environmental cues. The most prevalent mechanism is diffusion-controlled release, where factors diffuse through scaffold pores or polymer matrices into surrounding tissues, necessitating scaffolds with high porosity [99]. Another common approach is degradation-controlled release, in which the release rate of factors correlates directly with the scaffold’s degradation rate [100]. Advanced strategies further enhance release precision, such as stimuli-responsive systems that activate under specific triggers—for example, pH-sensitive polymers (e.g., sulfonic acid-modified polymers) or enzyme-cleavable linkages for external stimuli (e.g., matrix metalloproteinase (MMP)-cleavable peptide linkages), and temperature-responsive materials like poly(N-isopropylacrylamide) for internal thermal cues [101,102,103]. Additionally, sequential release platforms utilize layered or core–shell architectures to deliver multiple factors in a timed sequence (e.g., prioritizing VEGF for vascularization before releasing BMP-2 for bone formation), thereby aligning therapeutic action with physiological repair stages [104].

The primary challenges in sustained-release systems lie in mitigating burst–release effects, preserving factor bioactivity, ensuring scaffold stability, and addressing various potential side effects.

## 3. Clinical Application Scenarios of Bone Scaffolds

From a clinical perspective, the application of bone scaffolds generally addresses several practical clinical needs, including the filling of bone defects and the functional reconstruction of bone tissue.

Firstly, the most common application is to treat traumatic bone defects, that is, the repair of large segmental bone defects and comminuted fractures, which is the most suitable clinical scenario for the application of bone scaffolds [105]. Using bone scaffolds to fill the missing bone tissue is straightforward, and the technology is relatively simple and easy to promote. According to relevant reports, high-energy traumas (such as car accidents and gunshot wounds) often lead to large-segment bone defects (>5 cm) [106]. Traditional autologous bone grafts have limitations such as donor-site complications (with an infection rate of 15–30%) and insufficient bone mass [107]. In addition, due to the difficulty of reducing bone fragments in comminuted fractures, it is prone to delayed union or non-union (with an incidence rate of approximately 5–10%) [108]. Commonly used bone scaffold materials within clinical practice include composite scaffolds and alloy scaffolds, such as PLA scaffolds and titanium-alloy scaffolds [109]. With the use of technologies like 3D printing, the precise matching of the defect area can be achieved [110]. However, there are still problems such as poor mechanical matching and the risk of infection. For example, large-segment weight-bearing bones (such as the femur) require the scaffold to have a much higher compressive strength [111]. Most existing materials rely on metal reinforcement, but metal materials inevitably limit degradability. At the same time, open wounds are likely to lead to bacterial colonization on the surface of the scaffold, often requiring the integration of an antibacterial coating on the bone scaffold [112].

Another clinical application scenario is osteonecrosis and degenerative diseases, mainly including avascular necrosis of the femoral head and osteoporotic fractures [113]. This type of application emphasizes the therapeutic ability and functional properties of bone scaffolds. Commonly used bone scaffolds in clinical applications include bisphosphonate-loaded β-TCP scaffolds and strontium-doped bioactive glass (Sr-BG) scaffolds [114,115]. The former, due to its property of sustained-release drug-loading, is considered to have a certain inhibitory effect on osteoclast activity, and its effectiveness in patients with early-stage avascular necrosis of the femoral head has been confirmed in a multi-center clinical study [116,117]. However, these bone scaffolds that are highly adapted to local needs also have some drawbacks, including the potential problem of osteoblast inhibition caused by excessive bisphosphonates and the insufficient mechanical strength of the bone scaffold after degradation [118].

There are also some other clinical application scenarios, including maxillofacial and cranial bone repair, spinal fusion, joint replacement, etc. These mainly target the application of local bone scaffolds with special requirements for three-dimensional structures. The main challenges include the impact on the tissues surrounding the bone tissue (such as changes in mechanical structure, infection, and growth requirements) [119].

The clinical application of bone-defect repair and functional reconstruction has shifted from “structural substitution” to “biological function regeneration”, but the mechanical properties of materials, the coordinated release of multiple factors, and personalized manufacturing still remain the core challenges [120].

## 4. Clinical Translation and Challenges

At present, the research on bone repair scaffolds with nano-sustained-release factors is mainly conducted at the laboratory level. Only a few nano-sustained-release bone repair products have obtained FDA or CE certification, and they are mainly concentrated in the field of growth factors(Table 2). The following lists some of the products already on the market:

1. (Medtronic)’s collagen sponge scaffold, which is loaded with recombinant human BMP-2 (rhBMP-2), is used for spinal fusion and tibial fractures. It has a remarkable curative effect (a fusion rate of 95% vs. 85% for autologous bone grafting), but it requires a high dose of BMP-2 (4.2–12 mg), and is prone to side effects such as heterotopic ossification (with an incidence rate of 10–30%) and neuroinflammation [121];
2. (Stryker)’s BMP-7 and collagen complex is generally used for the treatment of non-union of bones. However, due to high costs and insufficient market competitiveness, it has gradually withdrawn from the mainstream market [122];
3. The following lists some nano-sustained-release bone scaffold systems that have started clinical trials:
  3.1. One study plans to enroll forty patients requiring the extraction of one hopeless tooth, followed by alveolar bone regeneration (ABR) and the placement of an endosteal implant. After exodontia, participants will be randomized and divided into two groups: in the experimental group, the socket will be grafted with rhBMP-2-BBM granules; in the control group, the sockets will be grafted with Bio-Oss granules and covered with porcine collagen barrier membrane;
  3.2. One trial concerned a process in which the surface of a 3D-printed titanium-alloy scaffold is coated with a biphasic sustained-release layer of VEGF/BMP-2, which is used for the repair of comminuted fractures. The bone density recovery rate reached 90% of the normal bone structure 12 months after surgery.
4. The following lists some scaffolds that have not yet undergone clinical trials:
  4.1. Gene-activated scaffold (GAM): PEI-modified mesoporous silica nanoparticles are used to deliver the Runx2 gene. In animal experiments, the volume of new bone has been shown to be three times higher than that of traditional scaffolds. The initiation of a Phase I clinical trial is planned for 2025;
  4.2. Intelligent response system: pH-sensitive PLGA microspheres are loaded with vancomycin, which can achieve on-demand drug release in an infected bone defect model, with a bacterial clearance rate of >99%.

The relevant information and data discussed in part Ⅶ are taken from the following website: https://clinicaltrials.gov/ (accessed on 4 February 2025).

## 5. Conclusions

Currently, as they are limited by the technical level required and the lack of data on the long-term use of the relevant materials, bone scaffolds with nano-sustained-release factors inevitably have potential safety issues, such as material toxicity and immunogenicity problems. The former mainly includes nanoparticle residues and degradation by-products; that is, the long-term retention of non-degradable materials (such as mesoporous silica) may trigger chronic inflammation or fibrosis, or the degradation products (such as lactic acid) of some synthetic organic substances (PLA, PLGA, etc.) may accumulate locally, even inhibiting osteogenesis. Immunogenicity comes from some biological carriers. For example, viral vectors (such as adenoviruses) and cationic polymers (such as PEI) are likely to activate the immune system, instead increasing the risk of treatment failure.

Another problem that cannot be ignored is that the scaffolds synthesized in the laboratory may not be completely consistent with the scaffolds produced in the factory assembly line, mainly because the assembly line requires a highly consistent production process. At present, the manufacturing process of bone scaffolds with appropriate properties that are capable of effectively loading and releasing nano factors is often complex, and the requirements for various parameters in the production process are strict, which will create huge pressure on the establishment of the production line. Therefore, when developing such scaffolds, researchers should take these issues into account. On the one hand, they need to simplify the manufacturing steps as much as possible, and on the other hand, they need clear and more stable production parameters (including temperature, humidity, etc.). As mentioned before, several scaffolds with biphasic release factors have been introduced. The standardized properties of the scaffolds may not be suitable for every patient. At the same time, unlike traditional metal internal fixators, these bone scaffolds cannot be autonomously improved to a certain extent through simple physical methods. For example, surgical tools such as scalpels and scissors are often used by surgeons to transform bone scaffolds. Some standardized scaffolds may not be suitable for some patients. Another issue that cannot be ignored is that the manufacturing process of these bone scaffolds with nano-sustained-release factors is far more complex than that of traditional scaffolds. This also puts huge pressure on the quality control of manufacturers and the supervision of regulatory authorities; for example, sterilization is a large problem that any implant cannot avoid. Most nano-sustained-release factors and their carriers cannot tolerate traditional high-temperature and high-pressure disinfection or ethylene oxide sterilization methods. This not only increases the difficulty of production and supervision but also directly leads to the production cost of these scaffolds being much higher than that of traditional products, causing a great socioeconomic burden.

## Figures and Tables

**Table 1:** Comparison for nano-sustained release bone scaffold fabrication technologies.

	Electrospinning	3D Printing	Self-Assembly	Nanocoating
Applicable Materials	Synthetic polymers (PLA, PCL), natural polymers (collagen, chitosan)	Thermoplastics (PLGA, PEG), ceramics (hydroxyapatite), metal composites	Peptides, liposomes, amphiphilic polymers, DNA nanostructures	Titanium alloys, bioceramics, polymer substrates; coating materials (PLGA, chitosan, bioactive glass)
Applicable Factors	Antibiotics (gentamicin), growth factors (BMP-2, VEGF), small-molecule drugs	Cells (stem cells), macromolecular proteins (collagen), drugs (bisphosphonates)	Genes (siRNA), enzymes, hydrophobic drugs (paclitaxel)	Antimicrobials (Ag nanoparticles), osteogenic factors (Ca^2+^/PO_4_^3−^ ions), anti-inflammatory drugs (dexamethasone)
Loading Capacity	High loading (intrafiber encapsulation), uneven distribution	Moderate loading (porosity-controlled release), high spatial specificity	Low loading (molecular-level encapsulation), uniform distribution	Low-medium loading (surface adsorption/covalent bonding), tunable release kinetics
Release Mechanism	Fiber network delays diffusion + material degradation synergy	Macropore architecture regulation + material gradient design	Intermolecular forces (H-bonding/hydrophobic interactions) forming nanocapsules	Surface coating chemisorption/physisorption + ion-exchange release
Mechanical Properties	High flexibility (5–50 MPa), low compressive strength	High strength (50–200 MPa), trabecular bone-mimicking capability	Poor mechanical strength (<5 MPa), requires reinforcement	Substrate-dependent (titanium >300 MPa), coatings enhance surface bioactivity
Degradation Control	Tunable via polymer blending (3–12 months)	Material-dependent (PLGA: 6 months; ceramics: non-degradable)	Dynamic responsive degradation (pH/enzyme-triggered)	Independent coating degradation (1–-6 months), decoupled from substrate
Clinical Potential	Soft tissue-bone interface repair (enthesis regeneration)	Personalized large-segment defect reconstruction (craniofacial surgery)	Targeted drug delivery (post-tumor resection filling)	Anti-infection/osseointegration in joint implants (hip revision surgery)
Current Challenges	Limited 3D structural complexity; dense fibers impede cell infiltration	Trade-off between high-resolution printing and bioactivity; insufficient vascularization	Scalability issues; poor in vivo stability	Long-term delamination risks; microcracks due to interfacial stress
Future Directions	Multi-nozzle heterogeneous fiber composites + microfluidic electrospinning	4D-printed shape-memory scaffolds + integrated vascular networks	Biomimetic mineralization for mechanical enhancement + stimuli-responsive assembly	Atomic Layer Deposition (ALD) gradient coatings + self-healing coatings
Summery: 1. Electrospinning and 3D printing suit high-dose sustained release, while self-assembly and nanocoating show promise for targeted delivery. 2. 3D printing allows customization for cortical or trabecular bone requirements; other techniques require composite reinforcement. 3. Electrospinning and nanocoating lead in translation, whereas self-assembly remains preclinical				

**Table 2:** Approval status and developmental potential of nano-sustained release bone repair scaffold products.

	Status of Approval	Future Therapeutic Potential	Key Technologies/Mechanisms	Main Challenges
collagen sponge scaffold	FDA approval (for spinal fusion and tibia fractures)	High fusion rate (95% vs. 85% autologous bone graft) but dose optimization to reduce side effects	Collagen sponge scaffold loaded rhBMP-2 and promoted osteogenesis through sustained release of growth factors	High dose BMP-2 (4.2–12 mg) causes heterotopic ossification (10–30%), neuroinflammation and other side effects
BMP-7 and collagen complex	Has obtained FDA/CE certification, and gradually withdrew from the mainstream market due to the lack of market competitiveness	Suitable for nonunion therapy and need to reduce costs to improve clinical accessibility	BMP-7 is complexed with collagen, which stimulates bone regeneration	The cost is high, and the advantage of the efficacy compared with autologous bone transplantation is not significant
rhBMP-2-BBM granules	Clinical phase II trial (alveolar bone regeneration test in 40 patients)	May replace the traditional Bio-Oss bone powder and shorten the implant implantation cycle	Bioactive particles loaded with rhBMP-2, promoting a local increase in Bdensity	Small sample test, long-term stability to be verified
VEGF/BMP-2 coated 3D printed titanium-alloy scaffold	Phase I/II clinical trial (fracture repair)	With personalized bone defect repair, the recovery rate of BMD reached 90% in 12 months	The biphasic slow-release layer (VE GF/BMP-2) on the surface of the titanium alloy stent jointly promotes angiogenesis and osteogenesis	Long-term coating stability and large-scale production feasibility are yet to be solved
Gene-activation scaffold (GAM)	Clinical Phase I is scheduled to start in 2025	In animal experiments, the volume of new bone reached 3 times that of traditional stent, which is suitable for the regeneration of large bone defects	PEI modified mesoporous silica nanoparticles to deliver the Runx 2 gene and enhance the osteogenic differentiation of stem cells	Gene-editing safety is controversial, and the large-scale production process is complex
Intelligent-responsive PLGA microspheres	Preclinical study (infectious bone defect model)	PH Triggered drug release, bacterial clearance rate > 99%, promising to replace systemic antibiotic therapy	PH Sensitive PLGA microsphere load vancomycin, and the infectious microenvironment triggers targeted drug release	The metabolic path in vivo is unknown and the release kinetics need to be optimized to match the bone repair cycle
Summery: 1. 3D printing combined with growth factor release (such as VEGF/BMP-2 coated titanium-alloy scaffold) has become the mainstream direction of personalized treatment. 2. GAM drives bone repair, from structural replacement to functional regeneration, spanning. 3. Marketed products face side effects (such as heterotopic ossification of InFuse^®^), while new technologies (such as intelligent response systems) need to solve large-scale production and long-term safety verification.				

## Data Availability

No new data were created or analyzed in this study. Data sharing is not applicable to this article.
